# Histopathological Profile of Different Breast Lesions: A Single-Center Observational Study

**DOI:** 10.7759/cureus.60408

**Published:** 2024-05-16

**Authors:** Eman H Ibrahim, Tamer A Ali, Shatha Sharbatti, May Kh. Ismail, Nazeerullah Rahamathullah, Sunil K Bylappa, Neema H Khalfan, Mariam E Mohammed, Alaa M Qasem, Mohamed A Hussein, Sameh M Ibrahim, Gehad A Bashir, Mosab F Alassal, Mohamed Sobhy, Asmaa A Dahy, Ahmed Abuelsoud

**Affiliations:** 1 Department of Pathology, Al-Azhar University, Cairo, EGY; 2 Department of Pathology, Gulf Medical University, Ajman, ARE; 3 Department of Urology, Al-Azhar University, Cairo, EGY; 4 Department of Community Medicine, Gulf Medical University, Ajman, ARE; 5 Department of Biomedical Sciences, College of Medicine, Gulf Medical University, Ajman, ARE; 6 Department of Laboratory, Thumbay University Hospital, Ajman, ARE; 7 Department of Clinical Sciences, College of Medicine, Gulf Medical University, Ajman, ARE; 8 Department of General Surgery, Saudi German Hospital, Ajman, ARE; 9 Department of Oncosurgery, El Salam Oncology Institute, Cairo, EGY; 10 Department of Urology, Sheikh Khalifa Medical City, Abu Dhabi, ARE; 11 Department of Vascular Surgery, Saudi German Hospital, Ajman, ARE; 12 Department of General Surgery, Gulf Medical University, Ajman, ARE; 13 Department of Plastic Surgery, Al-Azhar University, Cairo, EGY

**Keywords:** breast cancer, granulomatous mastitis, benign breast lesion, breast histopathology, breast fibroadenoma

## Abstract

Objective: To describe the histopathological pattern of different breast lesions among tissue specimens sent to our laboratory.

Method: A record-based study using a retrospective review of 255 histologically diagnosed breast biopsy reports in the histopathology department of authors from December 2016 to November 2021 was conducted. The specimens were collected from core biopsy, lumpectomy, and mastectomy. All data obtained were analyzed using the Statistical Package for the Social Sciences (SPSS) version 28 (IBM SPSS Statistics, Armonk, NY). Then, the findings were presented using text, tables, and charts.

Result: A total of 255 breast lesions were analyzed in this study. Most of the cases were benign (58.8%), followed by inflammatory lesions (21.6%), and malignant (19.6%). Fibroadenoma was the most prevalent benign lesion (36.7%), and most of the patients (70.9%) were in the age group of 20-39 years old. The most common inflammatory lesion was granulomatous mastitis (56.4%), and most of the cases were diagnosed in the age group of 30-39 years old. Invasive ductal carcinoma (IDC) was the most encountered histological type of carcinoma (62%). Of the malignant cases, 52% were diagnosed before the age of 50 years. Among these 50 cases, grade 2 was the most prevalent one (46%).

Conclusion: Benign breast lesions are more common than malignant breast lesions, and fibroadenoma is the most common benign subtype. Granulomatous mastitis is the most prevalent inflammatory breast lesion. About two-thirds of malignant cases are non-Arab. Invasive ductal carcinoma with no special type (NST) is the most common malignant subtype.

## Introduction

Breast tissue undergoes various hormonal and environmental influences over a person's lifetime, leading to a diverse range of pathological changes, which can include both benign and malignant lesions [1]. Increased use of fine-needle aspiration cytology and advanced imaging techniques play an important role in the preoperative evaluation of different breast lesions. However, in most cases, histopathological examination still has the upper hand in the differentiation between benign and malignant lesions [2]. Many studies have reported that benign breast lesions are the most prevalent disorders among breast biopsies. These changes as such are not life-threatening. Proper understanding and appropriate clinical, radiological, and pathological diagnosis of these disorders are important to give a clear explanation to the patient, rule out the possibility of breast cancer, and institute an appropriate treatment [3]. These breast changes are more common in females of childbearing age, peaking between the ages of 30 and 50 [4]. Inflammatory diseases of the breast are rare (accounting for less than 1% of breast symptoms) and are caused by infections, autoimmune disease, or foreign body-type reactions to extravasated keratin or secretions [5]. These lesions are important not only in terms of local symptoms and discomfort but also because many may mimic malignancy [6].

Breast cancer is the most common cancer among women worldwide [7]. According to the World Health Organization (WHO), there were an estimated 2.3 million women diagnosed with breast cancer, representing 11.7% of all cancer cases, and almost 685,000 deaths from breast cancer worldwide in 2020 [8]. This makes breast cancer the first or second leading cause of female cancer deaths in 95% of countries and the fifth most common cause of cancer death overall globally [9,10]. In the United Arab Emirates (UAE), breast cancer accounts for the most diagnosed cancer in both sexes with 21.4% and 38.8% in females of all ages according to the WHO in 2020 [11,12]. It was the leading cause of cancer death in 2019, with an estimated average of 11.6% of cancer deaths yearly [13]. The UAE has lower age-standardized rates of breast cancer incidence and mortality when compared to Western countries [14], as most cases occur in women younger than 50 years [15]. Bendardaf et al. [16] reported that the average age of women diagnosed with breast cancer in the Northern Emirates is 10 years earlier than in developed countries. Breast cancer is a heterogeneous disease that includes multiple entities associated with unique histological and biological characteristics, clinical manifestations, behaviors, and responses to treatment [17]. Adenocarcinoma is the most prevalent type of malignant breast tumor, accounting for almost 95% of all breast malignancies. This type begins as a carcinoma in situ in the ductal or lobular system, but most of them (>70%) will eventually break through the basement membrane and infiltrate the stroma when they are clinically identified. Based on morphological and molecular characteristics, invasive carcinoma can be classified into several clinically significant subclasses. It comes in a range of morphological forms, with roughly two-thirds falling under the category of invasive ductal carcinoma of no special type (IDC-NST). Morphologically, the remaining one-third can be divided into distinct histological categories [5]. Clinically, invasive breast cancer is classified according to the size of the primary tumor, lymph node status, and the presence of local and distant spread. At the morphological level, breast cancer is classified according to histopathological type and grade [18]. Histopathological types of breast cancer include classic lobular carcinoma, pleomorphic lobular carcinoma, medullary carcinoma, metaplastic carcinoma, apocrine carcinoma, mucinous carcinoma, cribriform carcinoma, secretory carcinoma, and neuroendocrine carcinoma [19].

The available data regarding breast cancer in women from the Middle East and North Africa (MENA), especially from the Northern Emirates region of the UAE, are inconsistent and scarce [15]. Limited data is available for histopathological patterns of breast lesions in the UAE. A previous study was done at University Hospital Sharjah between March 2016 and July 2018. The study showed that 91% of the cases were diagnosed as benign breast lesions. Among breast cancer cases, the most prevalent type was invasive-ductal carcinoma with hormone-positive receptor molecular subtype [16]. Although the previous study provided information on molecular subtypes of included breast cancer cases, little information was shown on grading parameters, and there was no mention of the different types of benign and inflammatory breast lesions. More studies are needed to highlight various clinical and pathological characteristics of breast lesions in the UAE. Such information will help in planning specific preventive strategies, including education, and directing clinical decision-making.

## Materials and methods

This study utilized a record-based approach, conducting a retrospective review of 255 breast biopsy records from the surgical pathology daybooks of the histopathology department at the authors' institute from 2016 to 2021. All specimens submitted to the histopathology laboratory (including core biopsy, lumpectomy, and mastectomy) were fixed in a 10% formalin solution, processed using a histokinette automated tissue processor, paraffin-embedded, and sectioned at 3-5 microns thickness using a microtome machine before staining with hematoxylin and eosin. The obtained results were analyzed with regard to age, sex, and lesion type. Histological classification of breast tumors was performed according to the WHO classification of breast tumors, fifth edition. Data analysis was conducted using the Statistical Package for the Social Sciences (SPSS) version 28 (IBM SPSS Statistics, Armonk, NY), and findings were presented through text, tables, and charts.

Inclusion criteria

All breast biopsy reports with a specific histopathological diagnosis within the study period were included.

Exclusion criteria

Breast biopsy reports with non-specific histopathological diagnosis or reports of improper breast lesion biopsy and none breast proper lesions were excluded.

## Results

During the five-year period (2016-2021), a total of 255 breast lesions were received in the histopathology department of Thumbay University Hospital Laboratory. The patients' age range was 13-73 years, and most cases (n = 93/255; 36.5%) were in the 30-39 years category. Among the 255 cases, 239 (93.7%) were female, while only 16 (6.3%) were male. According to histopathological examination, most of the cases were benign (n = 150/255; 58.8%), followed by inflammatory lesions (n = 55/255; 21.6%), and the rest was malignant (n = 50/255; 19.6%). In our study, more than half (n = 26/50; 52%) of malignant cases were diagnosed before 50 years old, whereas most non-malignant cases (85.5% of inflammatory and 85.3% of benign cases) were seen before 50 years old as shown in Table 1.

**Table 1:** Distribution of cases by age and type of lesion

Types of breast lesions (n = 255)	Different age group					P-value
	<20 years old (n = 13) (5.1%)	20-29 years old (n = 47) (18.43%)	30-39 years old (n = 93) (36.47%)	40-49 years old (n = 48) (18.8%)	≥50 years old (n = 54) (21.18%)	
Inflammatory (n = 55) (21.6%)	2 (0.78%)	9 (3.53%)	27 (10.58%)	9 (3.53%)	8 (3.14%)	<0.001
Benign (n = 150) (58.8%)	10 (3.92%)	33 (12.94%)	56 (21.96%)	29 (11.37%)	22 (8.63%)	
Malignant (n = 50) (19.6%)	1 (0.39%)	5 (1.96%)	10 (3.92%)	10 (3.92%)	24 (9.41%)	

Among the 55 inflammatory lesions, the most prevalent one was granulomatous mastitis (n = 31/55; 56.4%), followed by duct ectasia (n = 13/55; 23.6%), fat necrosis (n = 6/55; 10.9%), and epidermal inclusion cyst (n = 5/55; 9.1%). Most of the patients (n = 27/55; 49.09%) with inflammatory lesions were in the age group of 30-39, and only eight (14.55%) cases were reported after the age of 50 years (Table 2).

**Table 2:** Distribution of inflammatory breast cases among different age groups

Histopathological patterns of inflammatory breast lesions (n = 55)	Different age group				
	<20 years old (n = 2) (3.64%)	20-29 years old (n = 9) (16.36%)	30-39 years old (n = 27) (49.09%)	40-49 years old (n = 9) (16.36%)	≥50 years old (n = 8) (14.55%)
Duct ectasia (n = 13) (23.6%)	1 (1.82%)	2 (3.64%)	7 (12.73%)	1 (1.82%)	2 (3.64%)
Granulomatous mastitis (n = 31) (56.4%)	0 (0%)	4 (7.27%)	16 (29.09%)	6 (10.91%)	5 (9.09%)
Fat necrosis (n = 6) (10.9%)	0 (0%)	3 (5.45%)	2 (3.64%)	0 (0%)	1 (1.82%)
Epidermal inclusion cyst (n = 5) (9.1%)	1 (1.82%)	0 (0%)	2 (3.64%)	2 (3.64%)	0 (0%)

Among the 150 benign breast lesions, fibroadenoma was the most prevalent lesion (n = 55/150; 36.67%), followed by fibrocystic disease (n = 44/150; 29.33%), gynecomastia (9.33%), sclerosing adenosis (5.33%), intraductal papilloma (4.7%), columnar cell changes (3.3%), benign phyllodes (3.3%), lipoma (2.7%), atypical ductal hyperplasia (ADH) (2%), tubular adenoma (1.3%), lactating adenoma (1.3%), and pseudoangiomatous hyperplasia (0.7%). In the present study, most cases (n = 128/150; 85.3%) of benign breast lesions were diagnosed before 50 years, and most of the patients (n = 56/150; 37.3%) were in the age group of 30-39 years. Only 22 (14.7%) cases were reported after the age of 50 years (Table 3).

**Table 3:** Distribution of benign breast lesions among different age groups ADH: atypical ductal hyperplasia

Histopathological patterns of benign breast lesions (n = 150)	Different age group				
	<20 years old (n = 10) (6.67%)	20-29 years old (n = 33) (22%)	30-39 years old (n = 56) (37.33%)	40-49 years old (n = 29) (19.33%)	≥50 years old (n = 22) (14.67%)
Fibroadenoma (n = 55) (36.67%)	5 (3.33%)	18 (12%)	21 (14%)	4 (2.67%)	7 (4.67%)
Fibrocystic disease (n = 44) (29.33%)	0 (0%)	5 (3.33%)	17 (11.33%)	13 (8.67%)	9 (6%)
Gynecomastia (n = 14) (9.33%)	3 (2%)	4 (2.67%)	7 (4.67%)	0 (0%)	0 (0%)
Sclerosing adenosis (n = 8) (5.33%)	0 (0%)	1 (0.67%)	3 (2%)	3 (2%)	1 (0.67%)
Intraductal papilloma (n = 7) (4.67%)	0 (0%)	1 (0.67%)	2 (1.33%)	1 (0.67%)	3 (2%)
Lipoma (n = 4) (2.67%)	0 (0%)	1 (0.67%)	1 (0.67%)	2 (1.33%)	0 (0%)
ADH (n = 3) (2%)	0 (0%)	1 (0.67%)	2 (1.33%)	0 (0%)	0 (0%)
Columnar cell changes (n = 5) (3.33%)	0 (0%)	0 (0%)	1 (0.67%)	3 (2%)	1 (0.67%)
Pseudoangiomatous hyperplasia (n = 1) (0.67%)	0 (0%)	0 (0%)	1 (0.67%)	0 (0%)	0 (0%)
Benign phyllodes (n = 5) (3.33%)	1 (0.67%)	1 (0.67%)	0 (0%)	2 (1.33%)	1 (0.67%)
Tubular adenoma (n = 2) (1.33%)	1 (0.67%)	0 (0%)	0 (0%)	1 (0.67%)	0 (0%)
Lactating adenoma (n = 2) (1.33%)	0 (0%)	1 (0.67%)	1 (0.67%)	0 (0%)	0 (0%)

**Figure 1:**
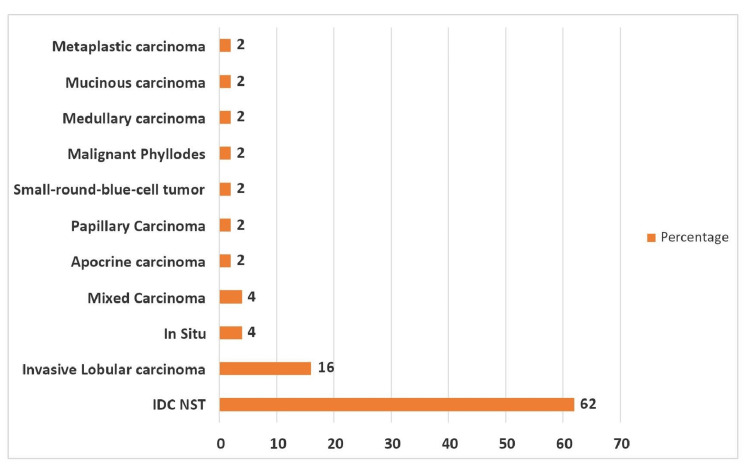
Histopathological patterns of malignant lesions (n = 50) IDC-NST: invasive ductal carcinoma of no special type

In this study, invasive ductal carcinoma of no special type (IDC-NST) was the most encountered type of carcinoma (n = 31/50; 62%). Invasive lobular carcinoma (ILC) came to a distant second (n = 8/50; 16%). The percentage of the rest of the rare cases is outlined in Figure 1.

In the present study, most cases (n = 26/50; 52%) of malignant breast lesions were diagnosed before 50 years, and only one case was reported at 16 years old (Table 4).

**Table 4:** Distribution of malignant breast lesions among different age groups IDC-NST: invasive ductal carcinoma of no special type

Histopathological patterns of malignant breast lesions (n = 50)	Different age group				
	<20 years old (n = 1) (2%)	20-29 years old (n = 5) (10%)	30-39 years old (n = 10) (20%)	40-49 years old (n = 10) (20%)	≥50 years old (n = 24) (48 %)
IDC-NST (n = 31) (62%)	1 (2%)	3 (6%)	6 (12%)	7 (14%)	14 (28%)
Invasive lobular carcinoma	0 (0%)	1 (2%)	1 (2%)	1 (2%)	5 (10%)
In situ carcinoma	0 (0%)	0 (0%)	0 (0%)	0 (0%)	2 (4%)
Mixed carcinoma	0 (0%)	0 (0%)	2 (4%)	0 (0%)	0 (0%)
Apocrine carcinoma	0 (0%)	0 (0%)	0 (0%)	0 (0%)	1 (2%)
Papillary carcinoma	0 (0%)	0 (0%)	0 (0%)	1 (2%)	0 (0%)
Small round blue cell tumor	0 (0%)	1 (2%)	0 (0%)	0 (0%)	0 (0%)
Malignant phyllodes	0 (0%)	0 (0%)	0 (0%)	0 (0%)	1 (2%)
Medullary carcinoma	0 (0%)	0 (0%)	1 (2%)	0 (0%)	0 (0%)
Mucinous carcinoma	0 (0%)	0 (0%)	0 (0%)	0 (0%)	1 (2%)
Metaplastic carcinoma	0 (0%)	0 (0%)	0 (0%)	1 (2%)	0 (0%)

**Figure 2:**
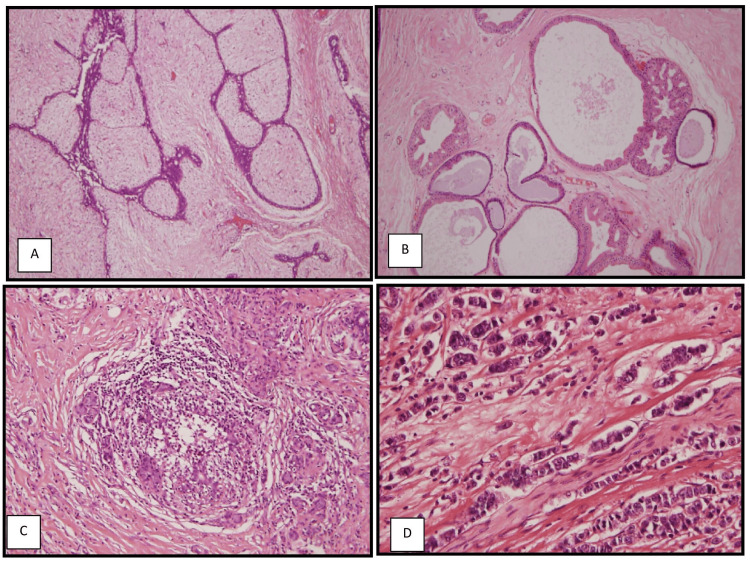
Most common subtypes of different breast lesions A: fibroadenoma, 200×; B: fibrocystic disease, 400×; C: granulomatous mastitis, 200×; D: IDC-NST, 400× IDC-NST: invasive ductal carcinoma of no special type

The malignant cases were graded using the Nottingham grading system. Among the 50 cases, grade 2 was the most prevalent (n = 23/50; 46%), while grades 1 and 3 represent 38% and 16%, respectively. Half of the cases (n = 13/26; 46%) before the age of 50 years old were grade 2. On the other hand, grades 1 and 2 represent most cases (n = 10/24; 41.7% for each one) after the age of 50 years old. In the current study, most of the breast lesions (n = 124/255; 48.6%) occurred in the left breast, followed by the right breast (n = 116/255; 45.5%). There were 15 bilateral cases (5.9%), and all of them were non-malignant. In this study, 46.8% (n = 96/205) of the non-malignant lesions were seen in the right breast, 45.8% (n = 94/205) were in the left breast, and only 7.3% (n = 15/205) were bilateral. Among the 50 malignant cases, 60% (n = 30/50) were seen in the left breast, 40% (n = 20/50) were in the right breast, and no malignant cases were bilateral. The histopathological features of the most common subtypes of different breast lesions in our study are reported in Figure 2.

## Discussion

Breast lesions are the leading cause prompting patients with breast concerns to seek surgical consultation. A thorough histopathological assessment of various types of breast biopsies is pivotal for accurate diagnosis, management, and follow-up of patients with diverse breast lesions [2]. This study aimed to determine the frequency of different histopathological patterns among a total of 255 biopsy tissues from various breast lesions presented at our histopathology department. These included core needle biopsies, incisional biopsies, excisional biopsies, lumpectomy, and mastectomy specimens. All patients, regardless of age or sex, with breast lesions were included. Notably, breast lesions are more prevalent among females than males. Out of the 255 breast lesions examined, 239 (93.7%) were identified in females, while only 16 (6.3%) were detected in males. Our results align with those of Kumbhakar and Talukdar (2021) [2], who observed a higher prevalence of breast lesions among females (99.66%) compared to males (0.34%). In our study, the majority of lesions were located in the left breast (48.6%), followed closely by the right breast (45.5%). Only 15 (5.9%) cases were bilateral, all of which were benign breast lesions. These findings are consistent with the observations of Yerakly and Tadele (2022) [1], who also noted that most breast lesions occurred in the left breast (51.1%), followed by the right breast (46.4%), with only three bilateral cases. Additionally, in our study, the overall mean age of patients was 38.64 years, with a standard deviation of ±12.3 and a wide age range of 13-73 years. These findings are comparable to those of a study conducted in Jamaica, where the overall mean age was 36.5 years, with a standard deviation of ±16.4 and an age range of 10-93 years [20].

The mean age in our study slightly surpasses that reported in Ethiopian, Nigerian, and Indian studies [1,21,22], where the mean ages were 34.96, 34.95, and 31.8 years, respectively. Several prior studies have noted a higher prevalence of benign breast lesions compared to malignant neoplasms. In our investigation, out of 255 cases, 205 (80.4%) were classified as non-malignant breast lesions, with 150 (58.8%) cases being benign breast lesions and 55 (21.6%) cases being inflammatory lesions. Conversely, 50 (19.6%) cases were identified as malignant neoplasms. The frequency of benign breast disease observed in our study aligns with findings from two studies conducted in India (76.60% and 72.80%) [2,23], as well as a study in Uganda (75%) [24]. A lower frequency of benign breast lesions was reported in a study conducted in Ethiopia (51%) [1], Nigeria (55.7%) [21], and India (50.4%) [25].

In our study, the most common age group for non-malignant (benign and inflammatory) breast lesions was between 30 and 39 years (40.5%), with a secondary peak age group of 20-29 years (16.8%). These findings are consistent with a study conducted in Nepal, where the peak age for benign breast lesions was between 31 and 40 years (40%), with a second peak of 21-30 years (32%) [26]. Similarly, Kapoor et al. (2020) [3] observed in their study that the age group of 30-49 years had the highest incidence of benign breast diseases (62.85%). In contrast to our findings, a study in India revealed that the first peak age for benign breast lesions was 21-30 years (37.9%), with a second peak age of 11-20 years (35.7%) [5]. Another study conducted in India also indicated that the peak age for benign breast lesions is 21-30 years (39.4%) [2]. However, our results are not consistent with a study conducted in Ethiopia, which found that the common age group for benign breast lesions was between 21 and 30 years (40%), with a secondary peak age group of 11-20 years (31.7%) [1].

In our study, fibroadenoma emerged as the most common benign breast lesion (n = 55/150; 36.7%), with the majority of patients presenting between the ages of 30 and 39 years (21 cases), followed by the age group of 20-29 years (18 cases). Additionally, fibrocystic disease represented the second most common benign breast lesion (n = 44/150; 29.33%), with the highest incidence in the age group of 30-39 years (17 cases), followed by the age group of 40-49 years (13 cases). This pattern was also observed by Kapoor et al. (2020) [3], who reported a higher prevalence (51.42%) of fibroadenoma and identified fibrocystic disease as the second most common benign breast lesion (22.85%) in their study. They also found that most cases of fibroadenoma occurred in the age group of 20-29 years, while cases of fibrocystic disease predominantly occurred in the age group of 40-49 years [3]. Our findings align with Yerakly and Tadele [1], who identified fibroadenoma as the most common benign lesion, accounting for 54.2% of benign cases. This observation is also supported by a study conducted by Tiwari and Tiwari [27], where fibroadenoma was the predominant lesion in benign breast disease, with fibrocystic disease being the second most common (25.7%) benign breast lesion. Similarly, Kapur et al. [28] noted that fibroadenoma comprised 41.7% of all benign breast diseases, with most patients presenting between 11 and 20 years, followed by 21-30 years of age [28]. They also found that fibrocystic disease was the second most common breast disease, accounting for 21.5% of benign breast lesions, with the highest incidence found in the age group of 31-40 years. Our results are further supported by a study by Bagale et al. [29], which reported fibroadenoma as the most common lesion (44.5%) among individuals aged. Similarly, a study in Nigeria revealed that fibroadenoma (63.1%) and fibrocystic change (19%) were the most common benign breast lesions, with the mean age of patients with fibroadenoma being 23.52 years [21].

In our study, inflammatory lesions were the second most common cause of non-malignant breast lesions, accounting for 21.6% (n = 55/255) of cases, with the majority of patients (n = 27/55; 49.1%) falling into the age group of 30-39 years, and only eight (14.5%) cases reported after the age of 50. This finding is consistent with Yerakly and Tadele [1], who found that the second most common breast lesion in their study was inflammatory breast lesions, accounting for 10.2% of breast lesions, with the peak age being 31-40 years [1]. Similarly, studies in Yemen, India, and Pakistan reported similar findings, albeit with lower frequencies ranging from 8% to 11.9% [30,31]. Among the 55 inflammatory lesions, the most prevalent was granulomatous mastitis (n = 31/55; 56.4%), followed by duct ectasia (n = 13/55; 23.6%), fat necrosis (n = 6/55; 10.9%), and epidermal inclusion cyst (n = 5/55; 9.1%). Conversely, Yerakly and Tadele [1] stated that the most common inflammatory lesion identified in their study was chronic non-specific mastitis (37.5%), followed by granulomatous inflammation (29.2%) [1].

Regarding malignant breast lesions, the majority of cases in our study (n = 24/50; 48%) were diagnosed in individuals above 50 years old, followed by a second peak in the age groups of 30-39 and 40-49 years (20% each), with only one case reported at 16 years old. Over half of all malignant cases in our study were diagnosed before the age of 50 years (n = 26/50; 52%). This trend is also observed in a study conducted in the UAE, which revealed that 52% of malignant cases were aged ≤50 years at the time of diagnosis [16]. Similar findings were reported in an Indian study, which noted that malignant breast neoplasms were predominantly observed in the age range of 41-60 years [1]. Our results are consistent with a previous study by Rasheed et al. (2014) [32]. In contrast to our study, two Ethiopian studies found that most malignant cases were between the age groups of 31 and 40 years [1,33]. In our study, 17 (34%) cases of malignant lesions were Arab, while non-Arab individuals accounted for two-thirds (66%) of cases.

Invasive ductal carcinoma of no special type (IDC-NST) was the most common type of carcinoma encountered (n = 31/50; 62%) in our study. Invasive lobular carcinoma (ILC) followed as the second most common type (n = 8/50; 16%), followed by mixed IDC and ILC, and in situ carcinoma (4% each). This finding is comparable to a study in Ethiopia, which reported IDC-NST as the most common (67%) malignant breast lesion, followed by invasive lobular carcinoma (12.2%) and mixed IDC and ILC (7.8%) [1]. Similar figures were found in an Indian study, where invasive ductal carcinoma of no special type constituted 71.2% of all malignant breast tumors [2]. This pattern is also reflected in findings from Sharjah, UAE, where IDC-NST was the most prevalent histopathological type encountered, accounting for 84% of all BRCA cases, followed by ILC and mixed IDC and ILC (5% each) [16]. Badowska-Kozakiewicz et al. (2017) [17] noted that IDC-NST accounted for 61.59% of all malignant cases, while ILC represented 10.52%. Similar findings have been reported from a study in the Kingdom of Saudi Arabia (KSA), which found that ductal carcinomas constituted the majority of cases, followed by lobular carcinomas (4%) and mixed ductal and lobular types (2%) [34].

In the current study, the majority of breast lesions (n = 124/255; 48.6%) occurred in the left breast, followed by the right breast (n = 116/255; 45.5%). There were 15 bilateral cases (5.9%), all of which were non-malignant. Among the non-malignant lesions, 46.8% (n = 96/205) were observed in the right breast, 45.8% (n = 94/205) were in the left breast, and only 7.3% (n = 15/205) were bilateral. Among the 50 malignant cases, 60% (n = 30/50) were observed in the left breast, 40% (n = 20/50) were in the right breast, and no malignant cases were bilateral. This finding aligns with a study conducted in Ethiopia, which reported the left breast as the most commonly involved side (52.2%) [1]. Similar findings were observed in a study conducted in Nigeria, where the left breast was the most commonly involved side in 51% of cases [35]. In contrast to our study, a study conducted in Addis Ababa reported that the right breast was the most involved side [33].

In our study, among the 50 malignant cases, grade 2 was the most prevalent (n = 23/50; 46%), while grades 1 and 3 accounted for 38% and 16%, respectively. Half of the cases (n = 13/26; 46%) diagnosed before the age of 50 years old were grade 2. Conversely, grades 1 and 2 represented most cases (n = 10/24; 41.7% each) diagnosed after the age of 50 years old. Similar findings were reported by Yerakly and Tadele (2022) [1], who found that 38% of tumors were grade 2, followed by grade 1 (28.6%), and grade 3 (22.1%). Studies in India, Nigeria, and Addis Ababa also showed the frequency of grade 2 tumors to be 59.5%, 50.6%, and 46.2%, respectively [33,36,37]. Our findings are in line with Badowska-Kozakiewicz et al. (2017) [17], who reported that the largest group of IDC-NST comprised grade 2 (53.96%), while grade 3 represented 28.98%. In contrast to our study, a study conducted in Nigeria found that the majority (78.7%) of IDC-NST were grade 3 at the time of presentation [35].

Limitations

The current study is a single-center study. In addition, the cases in this study did not have immunohistochemistry done to assess the hormone receptor status of malignant cases. Hormone receptor analysis is therefore not part of the result analyzed.

## Conclusions

Our study revealed that the most common breast lesions were benign, and the most common benign breast lesion was fibroadenoma. The current study also revealed that granulomatous mastitis is the most prevalent inflammatory lesion and invasive ductal carcinoma of no special type was the most common malignant subtype. More than half of our malignant cases were reported before 50 years, with most of them presenting as grade 2 tumors, and about two-thirds of these cases were non-Arab.
